# Losartan Slows Pancreatic Tumor Progression and Extends Survival of SPARC-Null Mice by Abrogating Aberrant TGFβ Activation

**DOI:** 10.1371/journal.pone.0031384

**Published:** 2012-02-14

**Authors:** Shanna A. Arnold, Lee B. Rivera, Juliet G. Carbon, Jason E. Toombs, Chi-Lun Chang, Amy D. Bradshaw, Rolf A. Brekken

**Affiliations:** 1 Division of Surgical Oncology, Departments of Surgery and Pharmacology, Hamon Center for Therapeutic Oncology Research, University of Texas Southwestern Medical Center, Dallas, Texas, United States of America; 2 Department of Medicine, Medical University of South Carolina, Charleston, South Carolina, United States of America; University of South Alabama, United States of America

## Abstract

Pancreatic adenocarcinoma, a desmoplastic disease, is the fourth leading cause of cancer-related death in the Western world due, in large part, to locally invasive primary tumor growth and ensuing metastasis. SPARC is a matricellular protein that governs extracellular matrix (ECM) deposition and maturation during tissue remodeling, particularly, during wound healing and tumorigenesis. In the present study, we sought to determine the mechanism by which lack of host SPARC alters the tumor microenvironment and enhances invasion and metastasis of an orthotopic model of pancreatic cancer. We identified that levels of active TGFβ1 were increased significantly in tumors grown in *SPARC-null* mice. TGFβ1 contributes to many aspects of tumor development including metastasis, endothelial cell permeability, inflammation and fibrosis, all of which are altered in the absence of stromal-derived SPARC. Given these results, we performed a survival study to assess the contribution of increased TGFβ1 activity to tumor progression in *SPARC-null* mice using losartan, an angiotensin II type 1 receptor antagonist that diminishes TGFβ1 expression and activation *in vivo*. Tumors grown in *SPARC-null* mice progressed more quickly than those grown in *wild-type* littermates leading to a significant reduction in median survival. However, median survival of *SPARC-null* animals treated with losartan was extended to that of losartan-treated *wild-type* controls. In addition, losartan abrogated TGFβ induced gene expression, reduced local invasion and metastasis, decreased vascular permeability and altered the immune profile of tumors grown in *SPARC-null* mice. These data support the concept that aberrant TGFβ1-activation in the absence of host SPARC contributes significantly to tumor progression and suggests that SPARC, by controlling ECM deposition and maturation, can regulate TGFβ availability and activation.

## Introduction

Pancreatic adenocarcinoma continues to carry a dismal prognosis with a 5 year survival rate of ∼5% in the United States [SEER]. A major clinical and therapeutic challenge for pancreatic cancer is the fact that the majority of patients present with advanced disease [1]. To combat pancreatic cancer in the high percentage of cases where the primary lesion has already spread beyond its local borders, it is imperative to understand the mechanisms driving invasion and metastasis.

SPARC (secreted protein acidic and rich in cysteine) is a glycoprotein that belongs to the matricellular class of proteins, a functional family of extracellular proteins involved in the regulation of extracellular matrix (ECM) deposition and remodeling. Although principally non-structural, matricellular proteins influence the structural integrity and composition of the ECM. After development, SPARC expression is limited to areas of high ECM turnover, such as bone and gut [2]. However, SPARC expression increases during wound-healing, angiogenesis and tumorigenesis [2], [3], [4], [5], [6]. *SPARC-null* (*SPARC−/−*) mice display characteristics suggestive of ECM defects, such as early cataract development, progressive osteopenia, lax skin and a curly tail [7]. In fact, collagen deposition and fibrillogenesis are disrupted in the lens capsule and dermis of *SPARC−/−* mice [8], [9]. These data suggest that SPARC is required for and mediates ECM deposition, and thus is critical for appropriate tissue remodeling.

In addition to its function in ECM assembly, SPARC directly binds to or indirectly regulates several growth factors including platelet-derived growth factor (PDGF), fibroblast growth factor (FGF), vascular endothelial growth factor (VEGF) and transforming growth factor β (TGFβ) [10], [11], [12], [13], [14]. Similar to SPARC, TGFβ is a multifunctional signaling protein implicated in wound-healing and fibrosis as well as tumor progression and metastasis [15], [16], [17]. In fact, data suggests that there is a reciprocal regulatory feedback loop between SPARC and TGFβ, whereby TGFβ induces the expression of SPARC and, in turn, SPARC modulates the expression and activity of TGFβ [13], [14], [18], [19], [20], [21], [22]. Additionally, SPARC may regulate growth factor signaling indirectly by affecting the deposition and composition of the ECM which, subsequently, controls the bioavailability of chemokines including TGFβ.

Previously, we demonstrated that in an orthotopic murine model of pancreatic adenocarcinoma, invasion and metastasis was increased in the absence of host SPARC. Consequently, tumor-bearing *SPARC−/−* mice experienced increased morbidity and decreased survival. In addition, we observed a clear reduction in the deposition of fibrillar collagens I and III, basement membrane collagen IV and the collagen-associated proteoglycan decorin in tumors grown in *SPARC−/−* mice. Paradoxically, tumors grown in *SPARC−/−* mice displayed a significant decrease in microvessel density and pericyte recruitment despite increases in invasion and metastasis. Enhanced vascular permeability and perfusion due to alterations in the vascular basement membrane led to decreased hypoxia in tumors established in the absence of host SPARC. Lastly, tumors grown in *SPARC−/−* mice displayed enhanced recruitment of fibroblasts and alternatively-activated (M2) macrophages [23] (Table 1).

**Table 1:** Pan02 Tumor-Associated Effects in *SP−/−* Mice.

Tumor Growth	Tumor Weight	NC
	Invasion & Metastasis	↑
	Survival	↓
Microvascular Properties	Microvessel Density	↓
	Pericyte Coverage	↓
	Vascular Basement Membrane Density	↓
	Permeability	↑
	Hypoxia	↓
Extracellular Matrix	Collagen Deposition	↓
	Collagen Fibrillogenesis	↓
	Decorin Content	↓
Infiltrating cell response	Fibroblasts	↑
	Neutrophils	NC
	Total Macrophages	↑
	M1 Macrophages	NC
	M2 Macrophages	↑

Table summarizing the tumor-associated effects previously analyzed in SPARC−/− (*SP−/−*) mice bearing orthotopic Pan02 tumors. Arrows indicate whether SPARC−/− mice displayed an increase (↑) or decrease (↓) compared to SPARC+/+ mice. NC, no change.

In the current study, we discovered that Pan02 tumors grown orthotopically in *SPARC−/−* mice have significantly increased levels of active TGFβ1 relative to those grown in *wild-type* (*SPARC+/+*) counterparts. To assess the contribution of excess active TGFβ1 on invasion, metastasis, survival, angiogenesis and immune modulation in the absence of host SPARC, TGFβ1 expression and activation was reduced by treatment with the angiotensin II type 1 receptor antagonist, losartan [24], [25]. We now report that losartan decreased invasion and metastasis, abrogated vasodilation, restricted permeability and regulated immune tolerance in tumor-bearing *SPARC−/−* mice, which effectively restored median survival to that of *SPARC+/+* mice. We provide evidence that the aberrant activation of TGFβ1 in the tumor microenvironment lacking stromal-derived SPARC contributes significantly to the phenotypic alterations observed during progression of orthotopic Pan02 tumors. We conclude that TGFβ1 is a significant driver of the increased tumor dissemination and decreased survival observed in tumor-bearing *SPARC−/−* animals.

## Results

### There is enhanced activation of transforming growth factor beta in tumors grown in SPARC-null mice

We showed previously that orthotopic pancreatic tumor growth of Pan02 cells is more invasive and metastatic in the absence of host SPARC and that this confers a decrease in the survival of *Sparc-null* (*SPARC−/−*) mice relative to *wild-type* (*SPARC+/+*) littermates [23], [26]. In addition, tumors grown in *SPARC−/−* mice displayed alterations in angiogenesis, ECM deposition and immune cell recruitment. Specifically, although microvessel density was reduced in tumors grown in the absence of host SPARC, the vascular basement membrane was compromised leading to enhanced permeability and decreased hypoxia. Collagen deposition and fibrillogenesis was also decreased. Furthermore, tumors grown in *SPARC−/−* mice displayed increased recruitment and activation of fibroblasts and alternatively-activated macrophages (M2). These alterations are summarized in Table 1.

**Figure 1:**
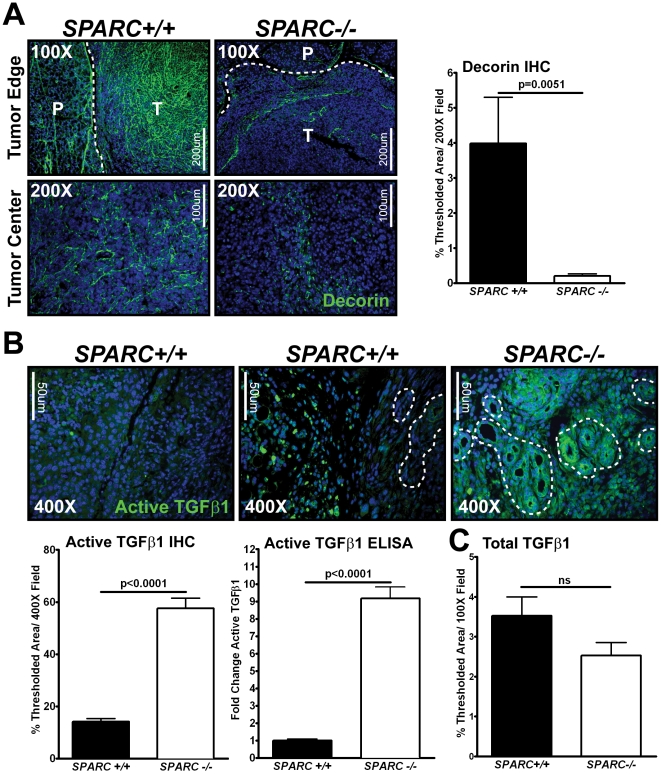
TGFβ1 and the TGFβ binding proteoglycan, decorin, are altered in tumors grown in the absence of stromal SPARC. Fluorescence immunohistochemistry was utilized to quantify the amount of active and total TGFβ1, as well as decorin in tumors grown in *SPARC+/+* and *SPARC−/−* mice (A–C). (A) Tumor sections were stained with antibody goat anti-decorin (green) and percent thresholded area quantified. DAPI (blue) marks cell nuclei. Images were taken at the tumor edge (top panels) and the tumor center (bottom panels). Total magnification is 100× (scale bar, 200 µM) and 200× (scale bar, 100 µM) as indicated. Dotted lines indicate the border between the tumor (T) and normal pancreas (P). (B) Tumor sections were stained with an antibody rabbit anti-TGFâ1 (green) specific for the active form and percent thresholded area quantified. DAPI (blue) marks cell nuclei. Total magnification is 400× (scale bar, 50 µM) as indicated. Dotted lines indicate spontaneous abnormal ducts arising from the Pan02 cells within the tumor. The amount of active TGFâ1 in tumor lysates was also measured using a commercial sandwich ELISA kit (G7591 Promega). Data represents two independent assays that are combined by normalizing all samples to *SPARC+/+* and recorded as fold change. 50 µg of total protein was loaded per well and samples were run in either duplicate or quadruplicate. (C) Total TGFβ1 protein within tumors was assessed with antibody rabbit anti-TGFβ1,2,3 and percent thresholded area quantified. All p-values were calculated with a Student's t-test. ns, not significant.

Although we discovered many changes in the tumor microenvironment of *SPARC−/−* compared to *SPARC+/+* animals, the underlying cause for increased invasion/metastasis and decreased survival remained elusive. An initial finding was that tumors grown in the absence of host SPARC displayed reduced deposition of the collagen-binding proteoglycan, decorin [23], presumably due to diminished deposition of collagen. We validated and quantified this result with fluorescence immunohistochemistry and found that decorin deposition in tumors grown in *SPARC−/−* mice was significantly reduced, not only within tumors, but at the tumor capsule (Figure 1A, p = 0.0051). Decorin and other ECM proteins have been shown to bind to, contribute to the bioavailability of, and modulate the activation of growth factors and cytokines [27]. We suspected that the diminished ECM associated with the absence of host SPARC might enhance tumor progression by altering growth factor localization and availability. In particular, it is established that decorin can directly bind and regulate the activity of TGFβ [28], [29], [30], [31]. Immunohistochemistry was performed utilizing antibodies directed against biologically active and total TGFβ1. The results revealed that, although there was no discernable change in total TGFβ1 (Figure 1C), there was a significant increase in active TGFβ1 in tumors grown in the absence of host SPARC (Figure 1B, p<0.0001). This was particularly apparent in spontaneous abnormal ducts in the Pan02 tumors. In addition, an ELISA specific for active TGFβ1 was performed on *SPARC+/+* and *SPARC−/−* tumor lysates. By ELISA, active TGFβ1 was increased by nine-fold in tumors grown in *SPARC−/−* compared to *SPARC+/+* mice (Figure 1B, bottom center, p<0.0001).

### Attenuation of TGFβ1 via losartan rescues survival of SPARC-null mice

**Figure 2:**
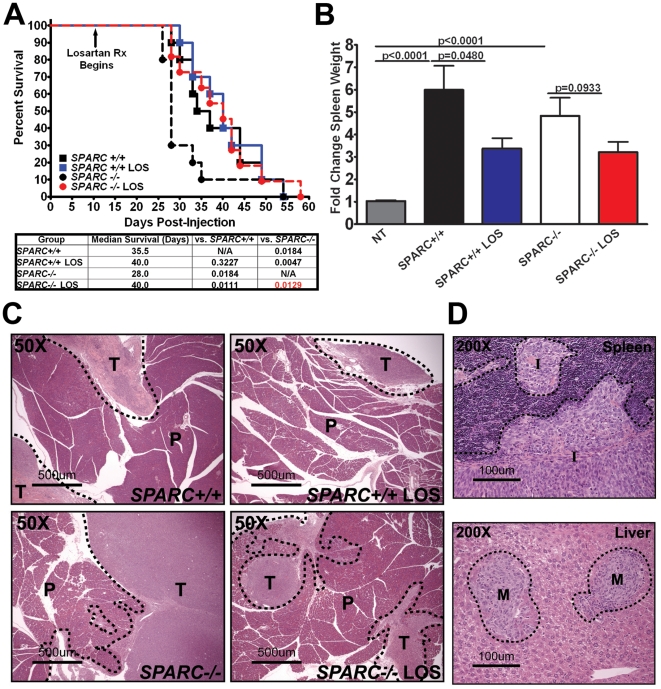
Losartan improves survival of tumor-bearing SPARC−/− mice. A–C) 1×10^6^ Pan02 cells were injected into the tail of the pancreas of *SPARC+/+* and *SPARC−/−* mice. Tumors were allowed to grow for 10 days prior to initiation of losartan therapy (600 mg/L) via drinking water *ad libitum* in 2% sucrose. A) Survival curve of *SPARC+/+* and *SPARC−/−* mice treated with losartan (LOS). The table lists the median survival and associated p-values calculated with the Gehan-Breslow-Wilcoxon test (n = 10/group). The survival curve is modified from Arnold et al 2010 [23]. B) Graph displays spleen weight and the fold change calculated based on the weight of spleens taken from non-tumor bearing (NT) mice. Significance was determined by a Student's t-test and all significant p-values are indicated. C, D) 1×10^6^ Pan02 cells were injected into the tail of the pancreas of *SPARC+/+* and *SPARC−/−* mice. Losartan therapy was given as above was initiated 24 hours after tumor cell injection and mice were sacrificed 28 days later. (C) Images of H&E stained primary tumor sections show residual pancreas and the primary tumor border of untreated and losartan-treated (LOS) *SPARC+/+* and *SPARC−/−* mice. Dotted lines demarcate the normal adjacent pancreas (P) from the primary tumor (T). Total magnification (50×) and scale bars (500 µm) are indicated. (D) H&E section of a spleen displaying disruption of the splenic capsule and local tumor invasion (I). Total magnification (200×) and scale bars (100 µm) are shown. Image of an H&E stained liver section revealing the common site for distant metastasis (M). Total magnification (200×) and scale bars (100 µm) are indicated.

Many of the phenotypic changes observed during tumor progression in *SPARC−/−* mice, such as enhanced vascular permeability, increased metastasis, decreased survival and altered immune tolerance, are consistent with elevated TGFβ activity [16], [17], [32], [33]. To assess the significance of TGFβ on the outcome of tumor progression, we performed a survival study in which tumor-bearing *SPARC−/−* and *SPARC+/+* animals were treated with losartan. Losartan, an angiotensin II type 1 receptor antagonist, is effective at inhibiting the expression and activation of TGFβ1 [25], [34], [35]. As previously reported, *SPARC−/−* mice succumbed to tumor burden more rapidly than their SPARC+/+ counterparts with a median survival of 28.0 days compared to 35.5 days (Figure 2A, p = 0.0184) [23]. Although targeting TGFβ1 with losartan had no significant effect on survival of *SPARC+/+* animals, the median survival of *SPARC−/−* mice was extended to 40.0 days by losartan therapy (Figure 2A, p = 0.0129). Losartan treatment delayed, but was unable to prevent, invasive and metastatic progression as at the time of sacrifice every animal exhibited extensive and aggressive tumor burden. However, losartan therapy did reduce the splenomegaly associated with Pan02 tumor progression (Figure 2B).

### Losartan reduces invasion and metastasis in SPARC-null mice

To assess the affect of losartan on tumor progression, local invasion and metastasis, an endpoint study was performed; whereby, losartan treatment was initiated the day following Pan02 tumor injection and all animals were sacrificed at four weeks, regardless of tumor burden or health status. This endpoint study revealed that losartan treatment was able to control local invasion and metastasis in *SPARC+/+* and *SPARC−/−* mice. Although there were no differences in tumor/pancreas weights between genotypes or treatment groups (data not shown), tumor growth was more confined to the pancreas in losartan-treated groups compared to untreated counterparts (Figure 2C). The effect of losartan on local invasion was apparent in the *SPARC−/−* mice, but not observed in *SPARC+/+* mice (Table 2). Losartan therapy reduced the number of *SPARC−/−* animals with signs of local invasion (invasion incidence) and the number of adjacent organs involved (Table 2). Local invasion occurred predominantly in the visceral adipose, abdominal muscle, intestine/mesentery and spleen (Figure 2D). *SPARC+/+* and *SPARC−/−* mice responded to losartan therapy with a reduction in the number of animals in each group with metastatic dissemination (metastatic incidence) and a diminution in the number of macroscopic metastatic lesions (Table 3). Metastasis occurred predominantly in the mesenteric lymph nodes and liver (Table 3). Importantly, although invasion and metastatic incidence and events were increased in *SPARC−/−* compared to *SPARC+/+* mice, as previously reported [23], [26], losartan normalized tumor progression in *SPARC−/−* mice to the equivalent of that observed in *SPARC+/+* counterparts (Table 2 and 3).

**Table 2:** Losartan reduces tumor invasion in SPARC−/− animals.

Invasion Incidence		
Group	n =	Total
*SPARC+/+*	10	8 (80%)
*SPARC+/+* LOS	8	8 (100%)
*SPARC−/−*	8	8 (100%)
*SPARC−/−* LOS	8	4 (50%)

Animals bearing orthotopic Pan02 tumors were treated with losartan as described in the methods. Local invasion was defined as tumor growth into adjacent organs with attachment to the primary tumor as determined by necropsy. Invasion incidence was defined as the number of animals in each group with signs of local invasion.

**Table 3:** The effect of losartan on Pan02 metastasis.

Metastatic Incidence										
Group	n =	Lung	Kidney	Liver	Spleen	Diaphragm	Intestine	I.P.	Other	Total
*SPARC+/+*	10	0	0	1 (10%)	0	2 (20%)	6 (60%)	4 (40%)	1 (10%)	7 (70%)
*SPARC+/+* LOS	8	0	0	0	1 (13%)	0	2 (25%)	2 (25%)	1 (13%)	4 (50%)
*SPARC−/−*	8	0	0	1 (13%)	1 (13%)	0	7 (88%)	0	1 (13%)	8 (100%)
*SPARC−/−* LOS	8	0	1 (13%)	0	0	0	6 (75%)	1 (13%)	0	6 (75%)

Total Metastatic Events											
Group	n =	Lung	Kidney	Liver	Spleen	Diaphragm	Intestine	I.P.	Other	Total	Mean
*SPARC+/+*	10	0	0	1	0	2	15	6	2	26	2.60
*SPARC+/+* LOS	8	0	0	0	1	0	4	2	1	8	1.00
*SPARC−/−*	8	0	0	2	2	0	23	0	1	28	3.50
*SPARC−/−* LOS	8	0	1	0	0	0	10	3	0	14	1.75

Mice bearing orthotopic Pan02 tumors were treated with losartan (600 mg/L). Therapy began immediately psot tumor cell injection and tumor burden was assessed 28 days later. Metastatic incidence was recorded as the number of mice bearing metastases in each organ listed in each treatment group. Total metastatic events/organ/group are also shown.

Therefore, local invasion and metastasis was reduced in SPARC−/− animals by treatment with losartan, which presumably accounts for the survival benefit observed (Figure 2A). The fact that levels of active TGFβ are elevated in tumors grown in *SPARC−/−* animals and that losartan decreases TGFβ activity supports the concept that excess TGFβ1 participates in the accelerated tumor progression and altered tumor microenvironment of orthotopic Pan02 tumors grown in the absence of host SPARC.

### Losartan reduces TGFβ pathway activation in SPARC-null mice

**Figure 3:**
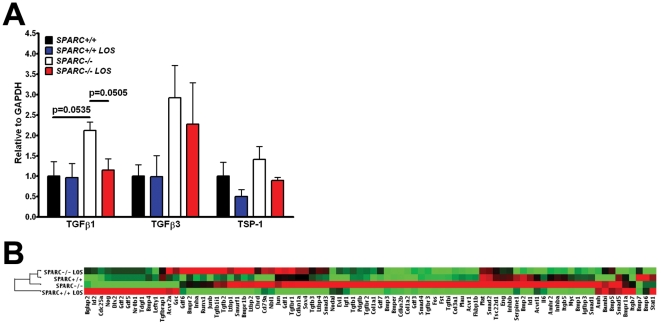
Losartan reduces TGFβ-induced gene expression in tumors from *SPARC−/−* mice. (A) The mRNA expression of TGFβ1, TGFβ3 and TSP-1 in tumors from *SPARC+/+* and *SPARC−/−* mice treated with losartan was assessed with taqman-based qPCR. n = 3/group and p-values were calculated with the Student's t-test. (B) Clustergram results from the RT^2^ Profiler™ PCR Array (PAMM-035; SABiosciences). Green indicates negative fold-regulation, whereas red indicates positive fold-regulation relative to six internal control genes.

Although losartan, an angiotensin II type 1 receptor antagonist, has been successfully utilized to inhibit the expression and activation of TGFβ1 in several animal models and in humans [24], [36], [37], [38], [39], we wanted to validate that TGFβ1 expression and downstream activation of TGFβ1 response genes were effectively inhibited by losartan in our model of pancreatic cancer. This was accomplished by performing real-time quantitative reverse-transcriptase polymerase chain reaction (qPCR). First, we analyzed the mRNA expression of TGFβ1, TGFβ2, TGFβ3 and thrombospondin-1 (TSP-1). Thrombospondin-1 is a matricellular protein that can function as a co-activator of latent TGFβ family members. TGFβ1 and TSP-1 are induced by the activation of angiotensin II type 1 receptor signaling and it is through reduction in TSP-1 expression that losartan is suspected to reduce the activation of latent TGFβ1 [40], [41]. Losartan decreased TGFβ1 and 3 mRNA expression in tumors from *SPARC−/−* animals to levels consistent with those observed in tumors from untreated *SPARC+/+* mice, although the decrease was not statistically significant possibly due to low sample number (Figure 3A). TGFβ2 mRNA was undetectable with the probes used (data not shown). The expression of TSP-1 was similar between tumors from *SPARC+/+* and *SPARC−/−* mice, but as expected there was a decrease in losartan-treated compared to untreated tumors in both genotypes (Figure 3A).

We next determined if losartan reduced TGFβ1-induced gene transcription. To broadly assess losartan effects on TGFβ1 signaling, we chose to perform the commercially available RT^2^ Profiler™ PCR Array (PAMM-035; SABiosciences, Frederick, MD). This array measures the level of mRNA of 84 TGFβ response genes. Consistent with the decrease in expression and activation of TGFβ1, many TGFβ response genes revealed altered expression (Table S1). As expected, since tumors grown in the absence of host SPARC harbor increased active TGFβ1, a majority of TGFβ target genes in tumors from *SPARC−/−* mice were elevated more than two-fold compared to tumors from *SPARC+/+* mice (Table S1, *SPARC−/−* vs. *SPARC+/+*). Relative to untreated counterparts, tumors grown in losartan-treated *SPARC+/+* and *SPARC−/−* animals showed more than a two-fold decrease of a majority of TGFβ target genes (Table S1, *SPARC+/+* LOS vs. *SPARC+/+* and *SPARC−/−* LOS vs. *SPARC−/−*). Most importantly, losartan treatment restored the mRNA expression profile of tumors grown in the absence of host SPARC to similar levels observed in untreated *SPARC+/+* mice (Figure 3B and Table S1, *SPARC−/−* LOS vs. *SPARC+/+*). Figure 3B shows the unsupervised hierarchical cluster analysis performed by the online RT^2^ Profiler™ PCR Array software provided by SABiosciences. Notice that the most closely associated TGFβ gene profiles are from tumors grown in *SPARC+/+* and losartan-treated *SPARC−/−* mice (Figure 3B). As a visual, Figure S1 shows heatmaps of the TGFβ response genes listed in Table S1, where each box represents a separate gene in the PCR array and each map is a relative comparison between two groups. Therefore, losartan effectively reduced the expression, activation and signaling of TGFβ1 during tumor progression in the absence of host SPARC.

### Losartan fails to restore angiogenesis and blood vessel maturation but leads to vasoconstriction and reduced permeability in tumors grown in SPARC-null mice

**Figure 4:**
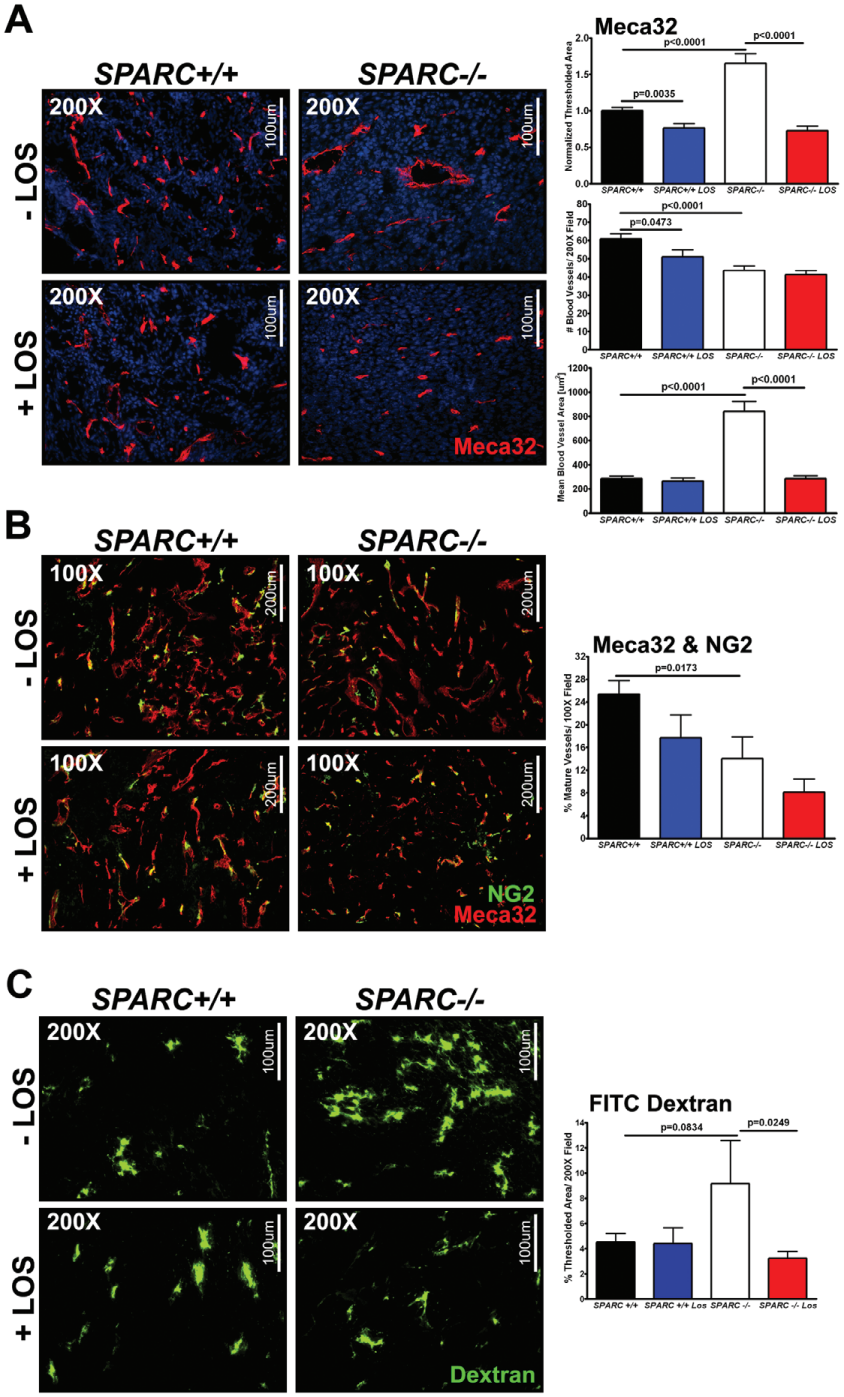
Losartan decreases vessel size but not effect microvessel density or pericyte recruitment in Pan02 tumors grown in *SPARC−/−* animals. Fluorescence immunohistochemistry was utilized to quantify microvessel density and pericyte recruitment in Pan02 orthotopic tumors grown in *SPARC+/+* and *SPARC−/−* mice treated with losartan (A–B). (A) Tumor sections were stained with rat anti-mouse endothelial cell Meca-32 (red) [64]. DAPI (blue) marks cell nuclei. The percent thresholded area normalized to untreated *SPARC+/+*, number of blood vessels and mean blood vessel area were quantified. Total magnification (200×) and scale bars (100 µm) are indicated. (B) Sections were stained with Meca-32 and rabbit anti-NG2. Percent mature vessels were calculated as the number of Meca-32 & NG2 colocalized vessels divided by the total number of Meca-32 positive vessels. Total magnification (100×) and scale bars (200 µm) are shown. C) *SPARC+/+* and *SPARC−/−* mice bearing orthotopic Pan02 tumors and treated with losartan were injected intravenously with fluorescein isothiocyanate-conjugated dextran (FITC-Dextran) (25 mg/ml) (2×10^6^ mw; D7137; Molecular Probes/Invitrogen) in 0.9% sterile saline at a dose of 200 µl/mouse. The fluorescent dextran was allowed to circulate 10 minutes before the mice were euthanized. Tissue was snap-frozen, sectioned (10 µm) and immediately analyzed by fluorescence microscopy. Percent thresholded area was quantified. Total magnification (200×) and scale bars (100 µm) are indicated. All p-values were calculated with the Student's t test.

We have reported previously that tumors grown in *SPARC−/−* mice demonstrated reduced microvessel density and pericyte recruitment, as well as increased permeability [23]. TGFβ1 can control angiogenesis, pericyte recruitment and blood vessel function during tumorigenesis [32], [42]. Therefore, we were interested in how excess TGFβ1 in the tumor microenvironment of *SPARC−/−* mice contributed to the aforementioned vascular changes. Microvessel density and pericyte recruitment were assessed by fluorescence immunohistochemistry. Staining with the pan endothelial cell marker, Meca-32, validated that microvessel density was significantly decreased in tumors grown in *SPARC−/−* mice compared to *SPARC+/+* controls (Figure 4A, p<0.0001). However, thresholded area was increased in the absence of host SPARC and many vessels appeared dilated (Figure 4A, p<0.0001). Therefore, the area of individual blood vessels or mean blood vessel area was assessed. Indeed, blood vessels were significantly larger and dilated in tumors grown in the absence of host SPARC accounting for the increase in thresholded area despite a decrease in microvessel density (Figure 4A, p<0.0001). Losartan treatment significantly reduced microvessel density in tumors from *SPARC+/+* mice (p = 0.0473) but did not affect microvessel density of tumors from *SPARC−/−* mice (Figure 4A). On the other hand, losartan significantly reduced the mean blood vessel area in tumors grown in the absence of host SPARC, essentially restoring blood vessel size to that found in tumors from *SPARC+/+* animals (Figure 4A, p<0.0001). Therefore, losartan treatment decreased microvessel density in tumors grown in *SPARC+/+* mice and constricted blood vessels in tumors from *SPARC−/−* mice.

Colocalization of Meca-32 and a pericyte marker, NG2, confirmed that pericyte recruitment was significantly diminished in tumors grown in *SPARC−/−* mice compared to *SPARC+/+* controls (Figure 4B, p = 0.0173). However, losartan therapy did not enhance pericyte coverage in tumors from *SPARC−/−* or *SPARC+/+* mice. In fact, although not significant, losartan further reduced pericyte recruitment in tumors grown in *SPARC−/−* and *SPARC+/+* mice. This is explained in part by the observation that losartan can inhibit pericyte migration [43].

In addition to alterations in microvessel density and maturity, tumors grown in *SPARC−/−* mice exhibited enhanced vascular permeability relative to tumors from *SPARC+/+* controls [23]. The permeability of fluorescein isothiocyanate-labeled dextran (FITC-Dextran) (2×10^6^ kDa) was determined for tumors grown in mice treated with losartan (Figure 4C). This experiment validated that tumors grown in the absence of host SPARC display increases in permeability to macromolecules (Figure 4C, p = 0.0834). More importantly, losartan treatment abrogated the increase in FITC-Dextran in tumors grown in *SPARC−/−* mice and restored permeability to levels equivalent to that observed in tumors grown in untreated *SPARC+/+* mice (Figure 4C, p = 0.0249).

Therefore, blockade of aberrant TGFβ1 activity with losartan in tumors grown in the absence of host SPARC reduces vasodilation and blood vessel permeability, but does not restore microvessel density.

### Collagen deposition and fibrillogenesis in tumors grown in SPARC-null mice is reduced by losartan

**Figure 5:**
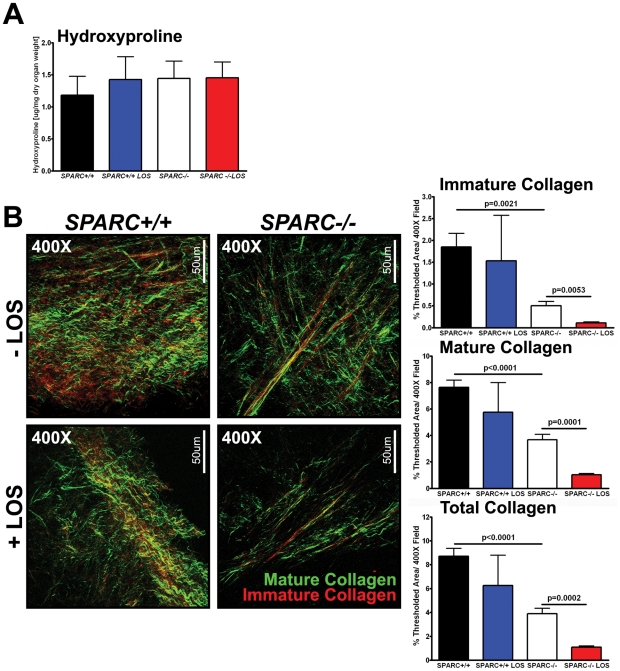
Losartan effect on collagen deposition and maturation. The amount of collagen production, deposition and maturation was assessed in Pan02 orthotopic tumors grown in *SPARC+/+* and *SPARC−/−* mice treated with losartan. (A) The amount of collagen produced and secreted within tumors was quantified by hydroxyproline analysis. (B) Collagen content and maturity was quantified by second harmonic generation (SHG). Frozen tumor sections (50 µm) were mounted in PBS. Collagen fibers within the tumor sections were excited at 900 nm to generate a SHG signal which was then detected at 450 nm. Both forward scattered signal, indicative of mature collagen (green) and backward scattered signal, indicative of immature collagen (red) was detected. Percent thresholded area of immature, mature and total collagen was quantified. Total magnification (400×) and scale bars (50 µm) are indicated. All p-values were calculated with the Student's t test.

Previous studies have shown that tumors grown in *SPARC−/−* mice have reduced collagen deposition and fibrillogenesis [23], [26], [44]. Although this was a consistent observation, the question still remained as to whether alterations in the collagen matrix influenced metastasis and/or survival of *SPARC−/−* mice bearing orthotopic Pan02 tumors. Due to the fact that losartan treatment improved survival and reduced metastasis in *SPARC−/−* mice, the next step was to determine if inhibiting TGFβ1 via losartan restored proper collagen deposition and maturation. Masson's trichrome staining confirmed that tumors grown in the absence of host SPARC display reduced collagen deposition (data not shown). However, losartan treatment failed to restore collagen deposition in tumors grown in *SPARC−/−* mice (data not shown). Furthermore, the amount of collagen production was unaffected by either genotype or losartan therapy as measured by hydroxyproline analysis (Figure 5A). When collagen deposition and maturation in orthotopic Pan02 tumors was quantified by second harmonic generation (SHG), it was found that collagen deposition and maturation decreased in tumors grown in the absence of host SPARC and that losartan augmented this effect resulting in an even further decrease in collagen deposition and maturation (Figure 5B). The deposition of immature (red), mature (green) and total collagen (both) were all significantly reduced in losartan-treated compared to untreated SPARC−/− mice (Figure 5B, p = 0.0053, p = 0.0001, p = 0.0002, respectively).

### Losartan fails to reduce the infiltration of alternatively-activated macrophages or myeloid derived suppressor cells

**Figure 6:**
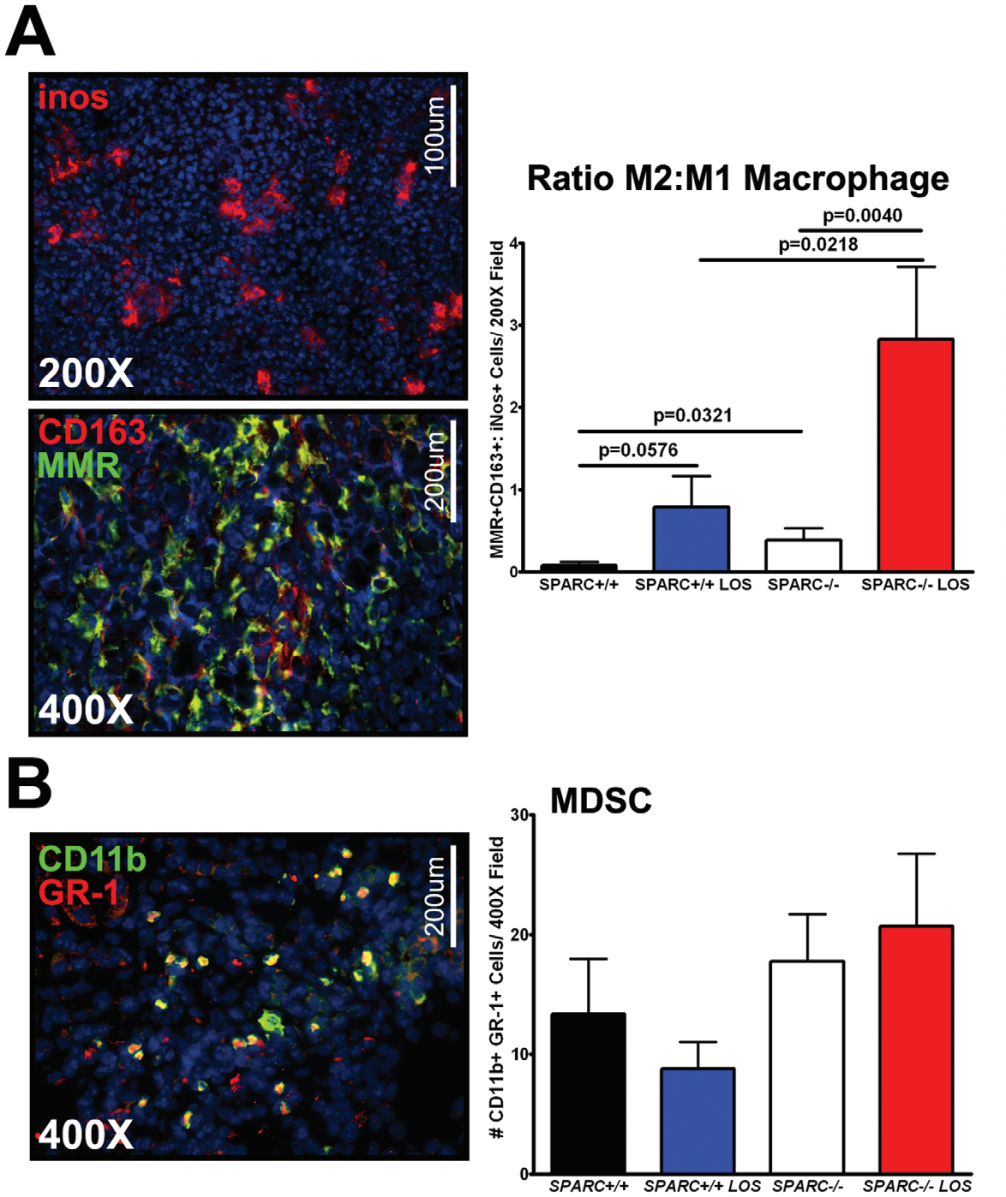
Losartan does not decrease macrophage and MDSC infiltration. Fluorescence immunohistochemistry was utilized to assess macrophage and myeloid derived suppressor cell (MDSC) recruitment in tumors grown in Pan02 orthotopic tumors grown in *SPARC+/+* and *SPARC−/−* mice treated with losartan (A–B). (A) Frozen tumor sections were stained with either antibody rabbit anti inos (red) for detection of M1 macrophage or antibody rabbit anti CD163 (red) and antibody rat anti MMR (green) for detection of M2 macrophage. DAPI (blue) marks cell nuclei. The number of inos positive cells and the number of CD163 and MMR double positive cells was counted and the ratio of M2 to M1 calculated. Total magnification (200×, 400×) and scale bars (100 µm, 200 µm) are indicated. (B) Frozen tumor sections were stained with antibodies FITC-conjugated rat anti CD11b (green) and CY3-conjugated rat anti GR-1 (red). DAPI (blue) marks cell nuclei. The number of CD11b and GR-1 double positive cells was counted. Total magnification (400×) and scale bars (200 µm) are indicated. All p-values were calculated with the Student's t test.

Previously, we identified that tumors grown in the absence of host SPARC exhibited an increase in macrophage recruitment and, more specifically, an increase in alternatively-activated macrophages (M2) [23]. Based on this result we hypothesized that the tumor microenvironment in *SPARC−/−* mice was “immune tolerant”, which could facilitate metastasis. As a result, we assessed macrophage recruitment and activation after losartan treatment. Immunohistochemistry for classically activated macrophages (M1) using iNos and M2 macrophages using CD163 and the mouse mannose receptor (MMR) validated that the ratio of M2 to M1 macrophage in tumors grown in the absence of host SPARC was significantly increased relative to those grown in *SPARC+/+* controls (Figure 6A, p = 0.0321). However, losartan therapy did not reduce the number of M2 macrophages in either genotype (Figure 6A). In fact, tumors grown in losartan-treated mice exhibited an even larger increase in the ratio of M2 to M1 macrophage (Figure 6A, *SPARC+/+* vs. *SPARC+/+* LOS, p = 0.0576; *SPARC−/−* vs. *SPARC−/−* LOS, p = 0.0040).

Myeloid-derived suppressor cells (MDSCs) have also been shown to activate tumor-associated immune tolerance [45]. Therefore, we performed fluorescence immunohistochemistry with antibodies targeting CD11b and GR-1, two markers that when colocalized serve to identify the MDSC population. Quantification of the number of CD11b^+^GR-1^+^ cells in tumor sections revealed that neither genotype nor losartan treatment significantly altered the recruitment of MDSCs (Figure 6B). Therefore, losartan restores survival and diminishes metastasis without reducing the infiltration of M2 macrophage and MDSC, cell populations known to participate in immunosuppression.

### Losartan reduces the activation of regulatory T-cells in tumors and spleens of SPARC-null mice

**Figure 7:**
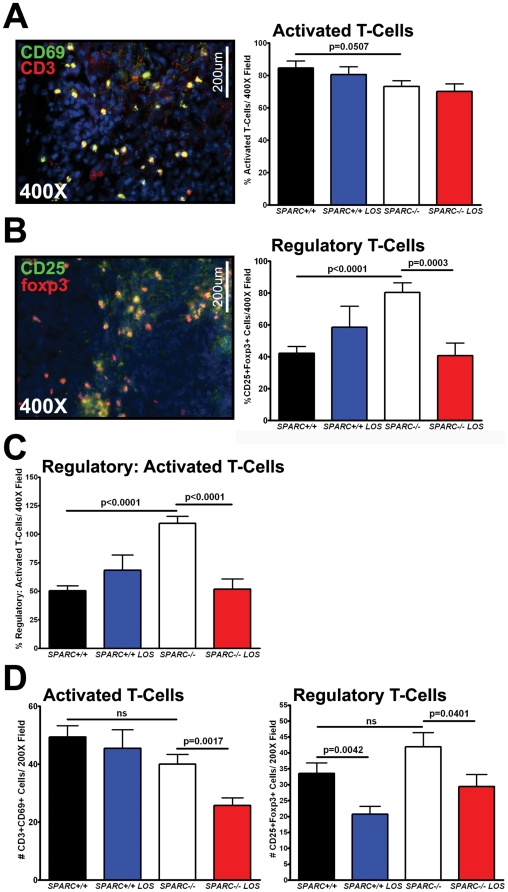
Losartan reduces Treg levels in Pan02 tumors grown in *SPARC−/−* mice. Fluorescence immunohistochemistry was utilized to assess the recruitment of activated and regulatory T-cells in Pan02 orthotopic tumors (A–C) or spleens (D) in *SPARC+/+* and *SPARC−/−* mice treated with losartan. (A) Frozen tumor sections were stained with antibody rat anti-CD3 (red) and hamster anti-CD69 (green) for detection of activated T-cells. DAPI (blue) marks cell nuclei. Percent activated T-cells was calculated by dividing the number of CD3 and CD69 double positive cells by the total number of CD3 positive cells. Total magnification (400×) and scale bars (200 µm) are indicated. (B) Frozen tumor sections were stained with rat anti-foxp3 (red) and rat anti-CD25 (green) for detection of regulatory T-cells. DAPI (blue) marks cell nuclei. Percent regulatory T-cells was calculated by dividing the number of foxp3 and CD25 double positive cells by the total number of CD3 positive cells. Total magnification (400×) and scale bars (200 µm) are indicated. (C) The ratio of regulatory T-cells to activated T-cells was calculated by dividing the number of CD3 and CD69 double positive cells by the number of foxp3 and CD25 double positive cells. Data was recorded as percent. (D) Frozen splenic sections were stained for activated and regulatory T-cells as described in A and B. The number of activated and regulatory T-cells was counted. ns, not significant. All p-values were calculated with the Student's t test.

A third population of immune cells implicated in immune tolerance are regulatory T-cells (Tregs) [46], [47], [48]. Tregs had not been assessed previously in this orthotopic model. Therefore, we performed fluorescence immunohistochemistry to assess the recruitment of activated and regulatory T-cells. Co-staining with the general T-cell marker, CD3, and the activated T-cell marker, CD69, allowed the quantification of percent activated T-cells or the number of CD3+CD69+ double positive cells divided by the total number of CD3+ cells. In general, the percent activated T-cells was not significantly different between the groups, although there was a marked trend towards decreased activated T-cells in tumors grown in the absence of host SPARC, regardless of losartan treatment (Figure 7A, p = 0.0507). On the other hand, the percent Tregs, assessed by colocalization of CD25 and foxp3, was significantly increased in tumors grown in *SPARC−/−* compared to *SPARC+/+* mice (Figure 7B, p<0.0001). More importantly, losartan treatment abrogated the increase of Tregs (Figure 7B, p = 0.0003). Since T-cell mediated immunosuppression is dependent on the balance between activated and regulatory T-cells, the ratio of regulatory CD25+foxp3+ T-cells to activated CD3+CD69+ T-cells was calculated (Figure 7C). Identical to the results for the percent Tregs, tumors from SPARC−/− mice had an increase in the ratio of regulatory T-cells to activated T-cells relative to those from SPARC+/+ mice and losartan therapy was able to neutralize this effect (Figure 7C, *SPARC+/+* vs. *SPARC−/−*, p<0.0001; *SPARC−/−* vs. *SPARC−/−* LOS, p<0.0001). T-cell recruitment and mobilization to the spleen was also determined (Figure 7D). Losartan treatment reduced the number of CD3+CD69+ double positive activated T-cells mobilized to the spleen in *SPARC−/−* mice (Figure 7D, p = 0.0017). In addition, although there were no changes in Treg recruitment to the spleens of *SPARC−/−* compared to *SPARC+/+* mice, losartan therapy significantly reduced the number of CD25+foxp3+ double positive Treg in both genotypes (Figure 7D, *SPARC+/+* vs. *SPARC+/+* LOS, p = 0.0042; *SPARC−/−* vs. *SPARC−/−* LOS, p = 0.0401).

Therefore, tumors grown in the absence of host SPARC display enhanced activation and recruitment of Tregs compared to those from *SPARC+/+* mice. Furthermore, losartan therapy was able to abrogate this increase and reduce the recruitment and mobilization of Tregs to the tumor and spleen. As a result, it is possible that losartan reduced metastasis and rescued survival of SPARC−/− mice by rebalancing immunosuppression within the tumor microenvironment.

### Pan02 cells in vitro and in vivo are undergoing epithelial-to-mesenchymal transition

An observation was made *in vitro* and *in vivo* that Pan02 cells exhibit many mesenchymal characteristics such as an elongated cell shape in culture with no signs of a cobblestone pattern characteristic of epithelial monolayers. This is important because TGFβ1 has been shown to have a dichotomous effect on tumor cell proliferation and migration depending on the epithelial versus mesenchymal nature of the cell [49]. Not only does TGFβ1 induce epithelial to mesenchymal transition (EMT) but, once a cell is mesenchymal, it no longer responds to TGFβ1 with immobility and quiescence [49], [50]. Instead, TGFβ1 induces the migration and proliferation of mesenchymal-like fibroblastoid tumor cells [49]. To determine if Pan02 cells have undergone EMT, we stained tumor sections with E-cadherin, an epithelial marker, and vimentin, a mesenchymal marker. The majority of the Pan02 tumors were negative for E-cadherin; however, spontaneous *de novo* ducts derived from Pan02 cells displayed robust E-cadherin expression (Figure S2A). These ducts, while abnormal, expressed E-cadherin that was appropriately localized to cell-cell junctions (Figure S2A, Zoom). This suggests that Pan02 cells are in the process of undergoing EMT, but still possess the ability to return to an epithelial-like state. Furthermore, Pan02 tumors displayed high expression of vimentin, which confirms that Pan02 cells display a mesenchymal-like phenotype (Figure S2B). As additional evidence, immunocytochemistry of Pan02 cells *in vitro*, reveals that β–catenin is localized to the nucleus (Figure S2C).

Therefore, the fact that Pan02 cells have undergone or are in the process of EMT, suggests that the excess TGFβ1 during tumor progression in *SPARC−/−* mice might directly stimulate their migration and subsequent metastasis.

### SPARC and TGFβ1 enhance Pan02 cell migration

**Figure 8:**
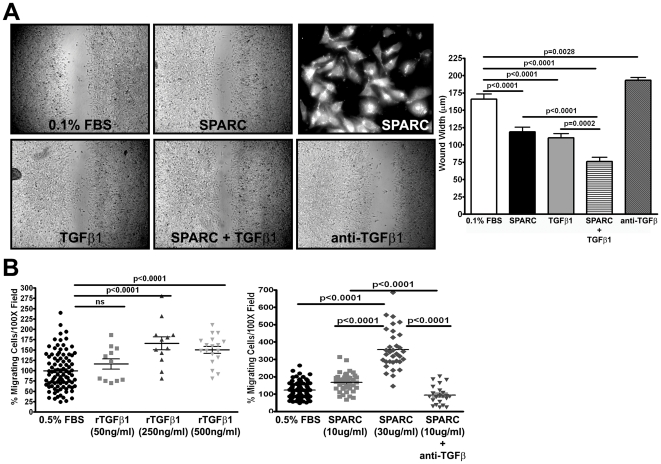
SPARC and TGFβ1 promote Pan02 migration. The ability of SPARC and TGFβ1 to control Pan02 cell migration *in vitro* was assessed. (A) A wound-healing assay was utilized to determine if SPARC and TGFβ1 could act concomitantly to enhance Pan02 migration. Cells were plated at a density of 1.5×10^5^ cells per well in 96-well plates and allowed to grow to 100% confluency. A scratch was made and cells allowed to migrate into the wound. Recombinant SPARC (10 µg/ml), recombinant TGFβ1 (250 ng/ml) and anti-TGFβ1,2,3 (10 µg/ml) were prepared in DMEM 0.1% FBS. The wound width (µm) was measured after 6 hours. Inset of immunocytochemistry with antibody goat anti-mouse SPARC reveals that Pan02 cells do express SPARC. (B) A transwell migration assay was performed as an additional means to assess SPARC and TGFβ1 effect on Pan02 migration, as well as, to determine if SPARC effect on Pan02 migration is TGFβ dependent. Cells were serum-starved overnight then seeded at 20,000 cells per 24-well 0.8 µm cell culture insert (BD Falcon). DMEM 0.1% FBS served as the chemoattractant. Recombinant SPARC (10 and 30 µg/ml) was added to the upper chamber, while recombinant TGFβ1 (50, 250 and 500 ng/ml) and anti-TGFβ1,2,3 (5 µg/ml) were added to the bottom chamber. Cells were allowed to migrate 24 hours at 37°C in 5% CO_2_. Migrated cells were fixed, stained and counted. Cell counts were normalized to DMEM 0.1% FBS and recorded as % migrating cells. ns, not significant. All p-values were calculated with the Student's t test.

To determine if Pan02 cell migration is influenced by SPARC and/or TGFβ1, *in vitro* scratch/wound assays were performed. Pan02 cells were treated with 0.1% FBS alone or in combination with recombinant SPARC (10 µg/ml), recombinant TGFβ1 (250 ng/ml), SPARC and TGFβ1, or anti-TGFβ1,2,3 (10 µg/ml). Wound width was measured six hours after wound initiation. Pan02 cells treated with SPARC or TGFβ1 exhibited a significant increase in wound closure or decrease in wound width relative to 0.1% FBS alone (Figure 8A, both p<0.0001). Furthermore, the combination of SPARC and TGFβ1 significantly accelerated wound closure compared to either treatment alone (Figure 8A, SPARC vs. SPARC+TGFβ1 p<0.0001; TGFβ1 vs. SPARC+TGFβ1 p = 0.0002). Lastly, neutralizing TGFβ signaling with an anti-TGFβ_1,2,3_ antibody significantly delayed wound closure compared to 0.1% FBS alone (Figure 8A, p = 0.0028). It is important to note that Pan02 cells express and secrete measurable amounts of SPARC (Figure 8A, top right panel). Therefore, if SPARC and TGFβ1 act concurrently to enhance Pan02 cell migration, then the secretion of SPARC by Pan02 cells *in vivo* may enhance the pro-migratory effects of excess TGFβ1. To validate that SPARC and TGFβ1 induce the migration of Pan02 cells, transwell migration assays were utilized. Pan02 cells migrated towards 0.5% FBS and were treated with recombinant SPARC (10 and 30 µg/ml), recombinant TGFβ1 (50, 250 and 500 ng/ml) or SPARC (10 µg/ml) in combination with anti-TGFβ1,2,3 (10 µg/ml). TGFβ1 dose-dependently enhanced Pan02 migration (Figure 8B, left graph). Likewise, SPARC also dose-dependently increased Pan02 migration (Figure 8B, right graph). Moreover, SPARC-induced migration was dependent on TGFβ1 because addition of the anti-TGFβ_1,2,3_ completely abrogated the effect of SPARC on migration (Figure 8B, right graph, SPARC (10 µg/ml) vs. SPARC (10 µg/ml)+anti-TGFβ_1,2,3_ p<0.0001).

Therefore, Pan02 migration is enhanced by SPARC and TGFβ1, whereby SPARC-induced migration is dependent on TGFβ1. This data provides plausible evidence that TGFβ1 in the tumor microenvironment is directly regulating the migration of Pan02 cells and stimulating their dissemination.

## Discussion

Our results provide evidence that enhanced metastasis and decreased survival of tumor bearing *SPARC-deficient* mice is a result of aberrant activation of TGFβ1. Inhibition of the expression and activation of TGFβ1 with the angiotensin II type 1 receptor antagonist, losartan, rescued survival of *SPARC−/−* mice challenged with Pan02 orthotopic tumors. It is likely that median survival of *SPARC−/−* mice was extended because local invasion and metastatic burden was reduced by losartan treatment.

Previously identified alterations in the tumor microenvironment between *SPARC+/+* and *SPARC−/−* mice were reassessed after losartan therapy. We found that vasodilation and blood vessel permeability are influenced by excess active TGFβ1 detected in tumors grown in the absence of host SPARC and, therefore, may contribute to metastasis and survival. On the other hand, decreased microvessel density and pericyte recruitment were not restored with losartan treatment. This is not altogether unexpected given the fact that the angiotensin system directly impacts vascular cells. Furthermore, considering that SPARC is required for proper collagen deposition and fibrillogenesis, it is not surprising losartan was unable to restore collagen deposition in tumors grown in the absence of host SPARC. In fact, the inhibition of TGFβ1 with losartan led to a further decrease in collagen deposition and maturation in tumors from *SPARC−/−* mice. We previously found that macrophage levels were enhanced in Pan02 tumors grown in *SPARC−/−* mice [23]. Losartan, which has been shown to inhibit inflammatory signaling in macrophages [51] promoted an increase in the M2∶M1 macrophage ratio in tumors grown in SPARC-deficient animals. Losartan treatment did however, negate the increase in Treg recruitment observed in tumors grown in the absence of host SPARC. This is likely due to inhibition of TGFβ, which in the tumor microenvironment promotes conversion of T cells to Treg cells [52]. Furthermore, Zhong et al [53] found that inhibition of TGFβR2 resulted in attenuation of TGFβ-mediated induction of Tregs and Treg mediated suppression of T-cell activation. Tregs contribute to tumor immune tolerance by neutralizing inflammatory responses and have been shown to correlate with worse clinical outcomes [48]. Thus, an imbalance in immunosuppressive and inflammatory cells provides another explanation for enhanced metastasis and decreased survival of *SPARC−/−* mice.

Losartan treatment had no effect on survival of wild-type mice bearing Pan02 tumors. Other reports suggest that losartan can slow the growth of Pan02 tumors and when used in combination with gemcitabine can result in potent inhibition of tumor growth [54]. Furthermore, Diop-Frimpong *et al.* [55] report that inhibition of collagen synthesis with losartan improves delivery of therapeutic nanoparticles. Neither of these reports document whether the anti-tumor mechanism of action of losartan is dependent upon inhibition of angiotensin II, rather both are consistent with an anti-TGFβ action of the drug. A confounding result is the slight increase in local invasion in wild-type mice treated with losartan (Table 2). The mechanisms underlying this are unclear; however, losartan therapy increased the M2∶M1 macrophage ratio in tumors grown in wild-type mice and M2 macrophages are known to promote tumor progression. Another intriguing possibility is that the increase in local invasion is a response to the anti-angiogenic activity of losartan. Inhibition of angiogenesis has been shown in some systems, including models of pancreatic cancer, to increase local invasion [56].

Although losartan was able to effectively reduce the expression, activation and signaling of TGFβ1, further validation is needed to prove that it is through the inhibition of TGFβ1 and not angiotensin II that tumor progression is reduced in *SPARC−/−* mice. Direct inhibition of TGFβ1 could be achieved with neutralizing antibodies to TGFβ1 or TGFβR2, [53], [57], [58] or small molecular weight inhibitors [59]. Survival, invasion, metastatic burden, vasodilation, blood vessel permeability and the immune profile would need to be reevaluated after treatment with a more selective TGFβ inhibitor and compared to the results of the losartan studies. LY2109761 has shown efficacy in murine models of pancreatic cancer [60]; however, to date antibody based strategies to inhibit TGFβ in pancreatic cancer have not been communicated.

It is clear that TGFβ1 is an important regulator of metastasis and survival in SPARC−/− mice bearing orthotopic pancreatic tumors. What is not completely clear is how losartan reduces metastasis and rescues survival of mice lacking SPARC. Several possibilities exist, including “normalization” of the vasculature by constricting the blood vessels and reducing permeability, as well as rebalancing the immune compartment by reducing regulatory T-cells. These effects could be mediated by inhibition of TGFβ. Another major possibility is that TGFβ1 is directly affecting the migration and dissemination of Pan02 cells and this is inhibited by losartan. At this point, we can only speculate as to why TGFβ1 is activated aberrantly in the absence of stromal SPARC. We hypothesize that alterations in the ECM such as decreased collagen deposition and reduced decorin, increase the bioavailable pool of latent TGFβ1, leaving more to bind and get activated at the cell surface. We have reported recently that SPARC can reduce TGFβ activity on the surface of pericytes [14]; we are investigating whether an analogous system is also in place on tumor cells. Expression of SPARC in human pancreatic adenocarcinoma is often lost due to promoter hypermethylation [61] and this correlates with the switch in TGFβ from a tumor suppressor to tumor promoter. It is therefore tempting to speculate based on the present results and our studies in SPARC-deficient animals [14], [23], [26] that SPARC is a central control point for regulation of TGFβ activity in the tumor microenvironment. Finally, therapy that includes losartan in pancreatic cancer patients that have lost tumor cell expression of SPARC is a potential strategy to control TGFβ-mediated tumor progression.

## Materials and Methods

### Tissue Culture

The murine pancreatic adenocarcinoma cell line (Pan02, also known as Panc02) was purchased from the Developmental Therapeutics Program, Division of Cancer Treatment and Diagnosis, National Cancer Institute (Frederick, MD), and grown in high glucose Dulbecco's Modified Eagles Medium (DMEM+GlutaMAX™-1, Invitrogen, Carlsbad, CA) supplemented with 5% fetal bovine serum (FBS, Life Technologies, Grand Island, NY). The Pan02 cell line was tested (Impact III PCR profiles; MU Research Animal Diagnostic Laboratory, Columbia, MO) and was found to be pathogen-free.

### Orthotopic Tumor Model

B6;129S-*Sparc^tm1Hwe^* mice were generated as described previously [62] and backcrossed into C57BL/6J a minimum of 10 generations. The mice were housed in a pathogen-free facility and experiments were conducted under a protocol (APN 0974-06-01-1) approved by the Institutional Animal Care and Use Committee of UT Southwestern Medical Center (Dallas, TX). All experiments were performed with *Sparc-null* (*SPARC−/−*) and *wild-type* (*SPARC+/+*) littermates. For injections, confluent cultures of Pan02 cells (>90% viable) were trypsinized, pelleted in DMEM 5% FBS, washed twice in phosphate buffered saline (PBS) and resuspended in 0.9% sterile saline (Sigma, St. Louis, MO). Tumor cells (1×10^6^) were injected directly into the tail of the pancreas to establish orthotopic tumors as previously described [26], [63]. For the survival study, individual mice were monitored daily and were euthanized when they displayed signs of tumor-associated morbidity such as excessive weight gain or loss, ascites, lethargy and/or distress. Bulk tumor growth was monitored through abdominal palpation. For the endpoint study, the entire cohort of mice was euthanized four weeks after tumor cell injection. The liver, heart, lung, kidney, brain, spleen and pancreas, including the tumor, were removed and weighed. Tumor could not be separated from the pancreas so tumor weights include residual pancreas. Metastasis and local invasion into surrounding organs was assessed macroscopically by visual examination. Suspected metastases were fixed, stained with H&E and verified as metastatic lesions histologically. Therefore, only visible macroscopic metastatic and locally invasive lesions were counted towards incidence and burden.

### TGFβ Inhibition with losartan, an Angiotensin II Type 1 Receptor Antagonist

Animals bearing orthotopic Pan02 tumors were treated with the angiotensin II type 1 receptor antagonist, losartan potassium (Cozaar, Merck, Whitehouse Station, NJ), shown to also inhibit the expression and activation of TGFβ. Therapy was given via drinking water *ad libitum* at a dose of 600 mg/L in 2% sucrose solution. The drinking solution was changed 3 times a week. Animals were randomized into treatment groups matched for age and sex. For the survival study, therapy began 10 days after tumor cell injection and continued the entire duration of the experiment. Therapy began immediately for the endpoint study and continued for 4 weeks until the entire group was sacrificed. Local invasion was defined as tumor growth into surrounding organs that remained attached to the primary tumor, while a nodule was considered a metastasis if it was located in a distant organ or clearly had no connection to the primary tumor.

### Immunohistochemistry

#### Fixed Tissue

Tissues were fixed in Methyl Carnoy's solution (60% methanol, 30% chloroform, 10% glacial acetic acid) and sent to the Molecular Histopathology Laboratory at UT Southwestern Medical Center (Dallas, TX) for paraffin-embedding and sectioning. The Molecular Histopathology Laboratory also performed staining with hematoxylin & eosin or Masson's trichrome. Tissue sections were deparaffinized and rehydrated in PBS containing 0.2% Tween-20 (PBSt) prior to staining.

#### Frozen Tissue

Tissues were snap-frozen in liquid nitrogen, embedded in optimal cutting temperature compound (OCT) (Tissue-Tek®), cut into 10 µm thick sections, air-dried overnight, fixed for 2 minutes in acetone and washed in PBSt prior to staining.

#### Fluorescence Detection

Deparaffinized and rehydrated sections were blocked in 20% AquaBlock (East Coast Biologics), incubated with primary antibody overnight at 40°C, incubated with fluorophore-conjugated (FITC or Cy3) secondary antibody (Jackson Immunoresearch) (1∶500) for 1–2 hours at room temperature and mounted with ProLong Gold Antifade Reagent with DAPI (Invitrogen).

#### Antibody Specifics

Primary antibodies used for IHC are listed in Supplemental Table S2. Antigen retrieval was performed, where indicated, by digesting with 20 µg/ml proteinase K for 5 minutes at room temperature.

### Imaging and Quantification

Tissue sections were analyzed with a Nikon Eclipse E600 microscope (Nikon, Lewisville, TX). Color images were captured with a Nikon Digital Dx1200me camera and Act1 software (Universal Imaging Corporation, Downington, PA). Fluorescence images were captured with a Photometric Coolsnap HQ camera and were captured randomly throughout the entire tumor, including the center and border, and under identical conditions including magnification and exposure time to allow quantification of signal intensities, object counts, percent thresholded area and colocalization with NIS Elements AR 2.30 software (Nikon). Images were thresholded to exclude background signal from secondary antibody alone. An average of ten images per tumor and a minimum of three tumors per group were used for each target.

### TGFβ1 enzyme-linked immunosorbent assay (ELISA)

Tumors were homogenized in lysis buffer (20 mM Tris-HCl pH 7.5, 150 mM NaCl, 1% Triton X-100) containing proteinase inhibitors (Complete Proteinase Inhibitor Cocktail Tablets, Roche, Indianapolis. IN). Tissue debris was pelleted and the resulting supernatant was used in subsequent analysis. Total protein was determined with a BCA protein assay (Pierce, Rockford, IL). An ELISA for active TGFβ1 (TGF beta 1 Emax ImmunoAssay System, Promega, Madison, WI) was performed on 50 µg of total protein per well according to the manufacturer's protocol. A minimum of 4 tumors per group was analyzed in duplicate. Data were normalized to *SPARC+/+*.

### Real-Time Quantitative Reverse-Transcriptase Polymerase Chain Reaction (qPCR)

#### RNA Isolation and Purification

RNA was isolated from tumors collected in the survival study utilizing TRIzol® (Invitrogen) reagent according to the manufacturer's protocol. Four milliliters of TRIzol® was used to isolate RNA from 100 mg of tumor. RNAse inhibitor (Roche) was then added to the isolated RNA and treated with DNAse (DNA Free Kit; Ambion, Austin, TX). The RNA was then further purified with the RNeasy Mini Kit (Qiagen, Valencia, CA). The samples were then eluted in RNAse/DNAse free water and utilized for subsequent cDNA synthesis.

#### Standard qPCR

Purified RNA, three samples per group, was reverse transcribed into cDNA using the iScript™ cDNA synthesis kit (Bio-Rad, Hercules, CA). Taqman probes for mouse GAPDH, transforming growth factor beta 1 (TGFβ1), transforming growth factor beta 3 (TGFβ3) and thrombospondin-1 (TSP-1) were purchased from Applied Biosystems (Foster City, CA). Real-time qPCR was performed with iTaq™ Supermix with ROX (Bio-Rad). The fold change was calculated as 2^−ΔCT^ where CT is the cycle threshold and ΔCT is the difference between the CT of the desired probe and the CT of GAPDH. Fold changes were then normalized to the *SPARC+/+* group for each probe.

#### TGFβ qPCR Array

The expression of 84 genes related to the TGFβ signaling pathway was assessed with the RT^2^ Profiler™ PCR Array (PAMM-035; SABiosciences, Frederick, MD). Synthesis of cDNA and real-time PCR was performed according to the manufacturer's protocol using the RT^2^ First Strand Kit (SABiosciences) and RT^2^ qPCR Master Mix (SABiosciences). Three RNA samples were pooled for each group prior to cDNA synthesis. Therefore, only one array was performed for each group. Cycle thresholds (CT) were uploaded to SABiosciences' online PCR array data analysis software allowing for the calculation of fold change/regulation and production of the clustergram and heatmaps. http://www.sabiosciences.com/pcr/arrayanalysis.php

### Fluorescent Dextran Permeability

Prior to sacrifice, mice were injected intravenously (tail vein) with fluorescein isothiocyanate-conjugated dextran (FITC-Dextran) (25 mg/ml) (2×10^6^ mw; D7137; Molecular Probes/Invitrogen, Eugene, OR) in 0.9% sterile saline at a dose of 200 µl/mouse. The fluorescent dextran was allowed to circulate 10 minutes before the mice were euthanized. Organs were removed, weighed, snap frozen, embedded in OCT and cut into 10 µm thick sections. FITC-dextran permeability was immediately assessed by fluorescence microscopy. A minimum of ten random photographs was taken of each tumor section, with an average of three animals per group. Results were recorded as mean percent thresholded area.

### Hydroxyproline Analysis

Mice were anesthetized with isoflurane and organs were removed and weighed as described before. Organs were then snap frozen, lyophilized, weighed (dry weight), pulverized and subjected to complete acid hydrolysis with 6 N HCl for 18 hours at 120°C. Each sample was then neutralized to pH 7 with 4 N NaOH. Chloramine T (1 ml) was added to 2 ml volumes of collagen sample and incubated at room temperature for 20 minutes. Ehrlich's Reagent (1 ml) (60% perchloric acid, 15 ml 1-propanol, 3.75 g p-dimethyl-amino-benzaldehyde in 25 ml) was added and samples were incubated at 60°C for 20 minutes. Absorbance at 558 λ was read on a spectrophotometer. Collagen was quantified as µg hydroxyproline per mg dry weight of starting material.

### Second Harmonic Generation Collagen Quantification

Frozen tumor sections (50 µm) were mounted in PBS and coverslipped. A Zeiss LSM510 META NLO using an Achroplan 40×/0.8 W objective lens (Zeiss, Roslyn, New York) was used to visualize the tissue sections. Collagen fibers within the tumor sections were excited at 900 nm with a Chameleon XR pulsed Ti:sapphire laser (Coherent, California) to generate a second harmonic generation (SHG) signal which was then detected at 450 nm. Excitation light was removed by a HQ 450 sp-2p filter (Chroma Technology, Vermont) and forward scattered signal, indicative of mature collagen, was detected with the transmitted light detector. A 680 nm short-pass dichroic mirror was utilized to remove backscattered excitation light. Backward scattered signal, indicative of immature collagen, was detected with a non-descanned detector placed at the illumination port of the wide-field epifluorescence light path. Two z-stack (10-step) images, at the areas of greatest collagen deposition, were taken for each tumor (n = 2) per group and the percent thresholded area quantified independently for every stack.

### Immunocytochemistry

Pan02 cells were plated on ibidi 8-well µslides (Applied Biophysics, San Diego, CA) at 10,000 cells per well. Cells were allowed to adhere overnight at 37°C 5% CO_2_. Cells were fixed in 10% formalin for 10 minutes at room temperature then permeabilized with 0.1% Triton X-100 for 5 minutes at room temperature. The slides were blocked for 1 hour in 20% Aquablock (East Coast Bio, North Berwick, ME) before incubation with primary antibodies rabbit anti-βcatenin (Cell Signaling Technology, Danvers, MA) or goat anti-mouse SPARC (R&D Systems, Minneapolis, MN) overnight at 4°C. Slides were incubated with secondary antibody (1∶500) for 1 hour at room temperature then mounted with DAPI ProLong® Gold antifade reagent (Invitrogen).

### Scratch/Wounding Assay

Cells were plated at a density of 1.5×10^5^ cells per well in 96-well plates and allowed to grow to 100% confluency. Serum containing media was removed, wells washed twice and replaced with serum-free DMEM. A scratch was then made down the center of each well with an extra long p10 pipette tip. The most uniform and consistent scratches were marked and used in the final analysis. Recombinant SPARC (10 µg/ml), recombinant TGFβ1 (250 ng/ml) and anti-TGFβ1,2,3 (10 µg/ml) were prepared in DMEM 0.1% FBS and added to the appropriate wells. Treatments were performed in duplicate. Images from the center of each well were taken at times 0 and 6 hours. The wound width (µm) was measured at a minimum of 20 locations along the wound for each replicate using NIS Elements AR 2.30 software. The initial wound width was used to verify consistency in scratches.

### Transwell Migration Assay

Cells were serum-starved overnight then plated in duplicate at 20,000 cells/well in 100 µl of serum-free DMEM in the upper chamber of a 24-well 0.8 µm cell culture insert (BD Falcon, San Jose, CA). The lower chamber of the 24-well plate received 300 µl DMEM 0.1% FBS as the chemoattractant. Recombinant SPARC (10 and 30 µg/ml) was added to the upper chamber, while recombinant TGFβ1 (50, 250 and 500 ng/ml) and anti-TGFβ1,2,3 (5 µg/ml) were added to the bottom chamber. Cells were allowed to migrate 24 hours at 37°C in 5% CO_2_. Cells were removed from the upper chamber with cotton swabs. Invading cells located on the under-side of the membrane were then fixed in 100% cold methanol 10 minutes and stained with hematoxylin and eosin. Membranes were removed and mounted on slides with Cytoseal (Thermo Fisher Scientific). A minimum of 10 images per replicate were taken at 100× total magnification and the number of invaded cells per field was counted. Cell counts were normalized to DMEM 0.1% FBS and recorded as % migrating cells.

### Statistical Analyses

Statistical analyses were performed using GraphPad Prism (GraphPad Software, San Diego, CA). Immunohistochemistry quantification and all *in vitro* assays were analyzed with the unpaired Student's t test where a p-value less than 0.05 was considered significant. The survival curves were analyzed with the Gehan-Breslow-Wilcoxon test.

## Supporting Information

Figure S1 **Heatmaps from the RT^2^ Profiler™ PCR Array displaying the TGFβ response genes listed in Table S1.** Each box represents a separate gene in the PCR array and each map is a relative comparison between two groups as indicated. Fold change log^2^ scales are shown below each heatmap.
(TIF)

Figure S2 **Pan02 cells have a mesenchymal-like phenotype.** Fluorescence immunohistochemistry (A–B) and immunocytochemistry (C) were utilized to assess the epithelial versus mesenchymal nature of Pan02 cells. (A) Frozen tumor sections were stained with antibody rabbit anti E-Cadherin (red) to mark epithelial cells. DAPI (blue) marks cell nuclei. Total magnification (200×) and scale bars (100 µm and 50 µm) are indicated. (B) Frozen pancreas and tumor sections were stained with antibody goat anti Vimentin (red) to mark mesenchymal cells. DAPI (blue) marks cell nuclei. Total magnification (200×) and scale bars (100 µm) are indicated. (C) Pan02 cells were stained with antibody rabbit anti β-catenin (red) to visualize its cellular localization. DAPI (blue) marks cell nuclei. Total magnification (100×) and scale bars (200 µm) are indicated.
(TIF)

Table S1 **The effect of losartan on TGFâ signaling pathways.** RT^2^ Profiler PCR Array (SA Biosciences) analysis of mRNA fold regulation of 84 TGFβ response genes in tumors from SPARC−/− and SPARC+/+ mice treated with losartan. Greater than negative two-fold regulation is indicated in *italics*, while greater than positive two-fold regulation is indicated in **bold** font.
(DOCX)

Table S2 **List of antibodies.**
(DOCX)
