# Supplementary figures and images for: Losartan Slows Pancreatic Tumor Progression and Extends Survival of SPARC-Null Mice by Abrogating Aberrant TGFβ Activation
Source: PLoS One. 2012 Feb 14;7(2):e31384. doi: 10.1371/journal.pone.0031384 (PMC3279359; doi:10.1371/journal.pone.0031384)

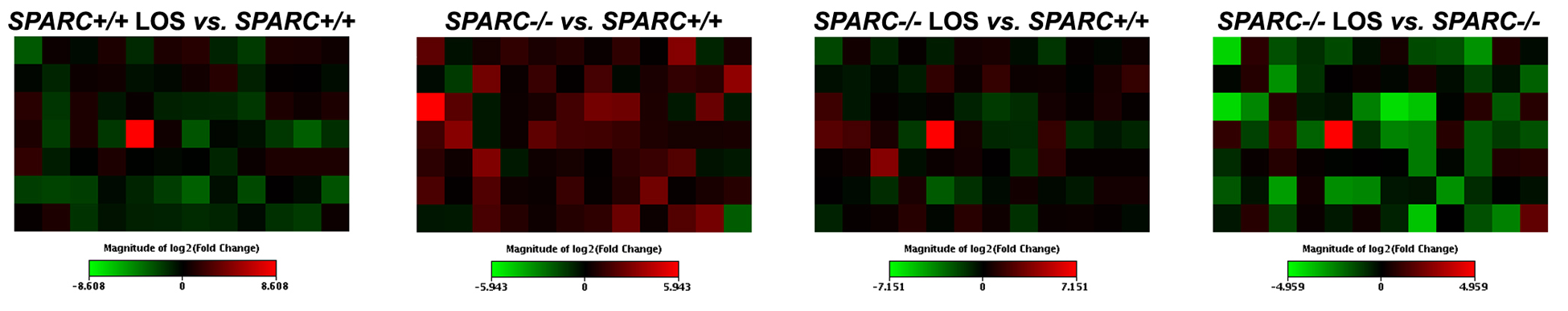

Supplement: Figure S1 — Heatmaps from the RT2 Profiler™ PCR Array displaying the TGFβ response genes listed in Table S1. Each box represents a separate gene in the PCR array and each map is a relative comparison between two groups as indicated. Fold change log2 scales are shown below each heatmap. (TIF) [file pone.0031384.s001.tif]

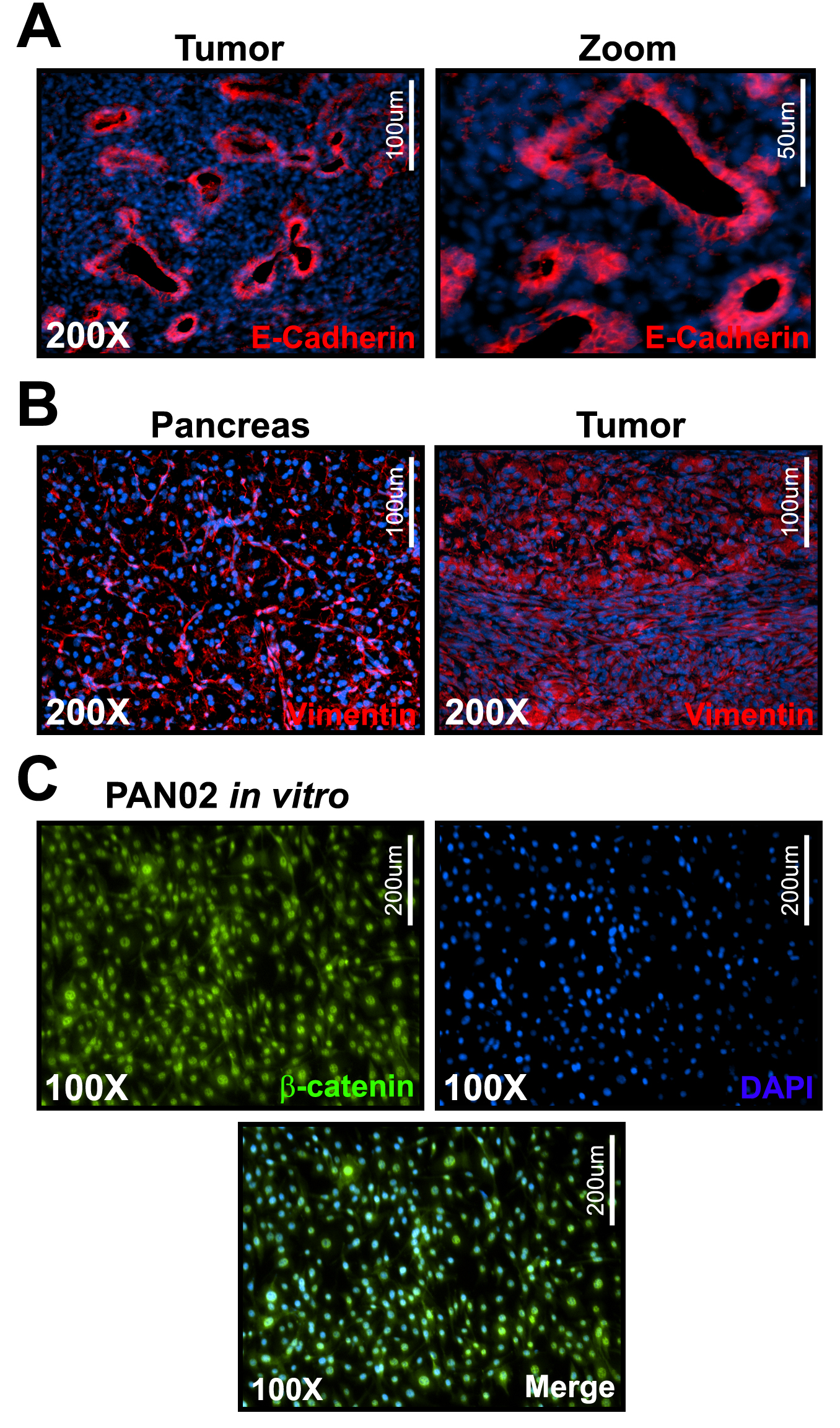

Supplement: Figure S2 — Pan02 cells have a mesenchymal-like phenotype. Fluorescence immunohistochemistry (A–B) and immunocytochemistry (C) were utilized to assess the epithelial versus mesenchymal nature of Pan02 cells. (A) Frozen tumor sections were stained with antibody rabbit anti E-Cadherin (red) to mark epithelial cells. DAPI (blue) marks cell nuclei. Total magnification (200×) and scale bars (100 µm and 50 µm) are indicated. (B) Frozen pancreas and tumor sections were stained with antibody goat anti Vimentin (red) to mark mesenchymal cells. DAPI (blue) marks cell nuclei. Total magnification (200×) and scale bars (100 µm) are indicated. (C) Pan02 cells were stained with antibody rabbit anti β-catenin (red) to visualize its cellular localization. DAPI (blue) marks cell nuclei. Total magnification (100×) and scale bars (200 µm) are indicated. (TIF) [file pone.0031384.s002.tif]
