# Supplementary material for: Losartan Slows Pancreatic Tumor Progression and Extends Survival of SPARC-Null Mice by Abrogating Aberrant TGFβ Activation
Source: PLoS One. 2012 Feb 14;7(2):e31384. doi: 10.1371/journal.pone.0031384 (PMC3279359; doi:10.1371/journal.pone.0031384)
Supplement: Table S1 — The effect of losartan on TGFâ signaling pathways. RT2 Profiler PCR Array (SA Biosciences) analysis of mRNA fold regulation of 84 TGFβ response genes in tumors from SPARC−/− and SPARC+/+ mice treated with losartan. Greater than negative two-fold regulation is indicated in italics, while greater than positive two-fold regulation is indicated in bold font. (DOCX) [file pone.0031384.s003.docx]

| **Table S1. The effect of losartan on TGFβ signaling pathways** | | | | |
| --- | --- | --- | --- | --- |
| **Fold Regulation** | | | | |
|  | **Relative to *SPARC+/+*** | | | **Relative to *SPARC-/-*** |
| **Gene Symbol** | ***SPARC+/+* LOS** | ***SPARC-/-*** | ***SPARC-/-* LOS** | ***SPARC-/-* LOS** |
| **Acvr1** | *-7.7947* | **4.4878** | *-3.8591* | *-17.3186* |
| **Acvr2a** | 1.3264 | -1.2968 | 1.3926 | 1.8059 |
| **Acvrl1** | -1.2488 | 1.4369 | *-2.0424* | *-2.9348* |
| **Amh** | 1.9419 | **2.1406** | 1.1382 | -1.8807 |
| **Amhr2** | *-2.5412* | 1.5347 | -1.7535 | *-2.6912* |
| **Bambi** | 1.9419 | 1.7888 | 1.4507 | -1.2331 |
| **Bglap2** | **2.4394** | 1.1884 | 1.5853 | 1.3340 |
| **Bmp1** | *-2.2447* | **2.1332** | -1.2819 | *-2.7344* |
| **Bmp2** | *-3.8464* | 1.0703 | *-2.7497* | *-2.9429* |
| **Bmp3** | 1.9419 | **8.4679** | 1.1382 | *-7.4397* |
| **Bmp4** | 1.8207 | -1.8113 | -1.1347 | 1.5963 |
| **Bmp5** | 1.3943 | 1.5168 | 1.3321 | -1.1386 |
| **Bmp6** | -1.1507 | -1.1867 | -1.2908 | -1.0877 |
| **Bmp7** | *-2.3030* | *-2.5263* | -1.5511 | 1.6287 |
| **Bmper** | 1.3027 | **6.0966** | -1.1698 | *-7.1317* |
| **Bmpr1a** | 1.4727 | 1.1567 | -1.6973 | -1.9633 |
| **Bmpr1b** | -1.4285 | **2.4470** | **2.5328** | 1.0351 |
| **Bmpr2** | -1.1781 | 1.0673 | 1.2507 | 1.1718 |
| **Cd79a** | 1.5226 | **2.9588** | **2.7165** | -1.0892 |
| **Cdc25a** | **2.3842** | -1.1243 | 1.2761 | 1.4347 |
| **Cdkn1a** | *-2.1265* | 1.5199 | 1.2993 | -1.1698 |
| **Cdkn2b** | 1.0729 | **2.2423** | -1.0198 | *-2.2867* |
| **Chrd** | 1.0684 | 1.9386 | 1.5765 | -1.2297 |
| **Col1a1** | -1.3375 | **10.1683** | **2.7967** | *-3.6357* |
| **Col1a2** | **2.5571** | **61.5207** | **3.2193** | *-19.1099* |
| **Col3a1** | *-3.3323* | **4.1039** | -1.5350 | *-6.2996* |
| **Dlx2** | 1.9419 | -1.4671 | 1.1382 | 1.6699 |
| **Eng** | -1.6466 | 1.2134 | -1.1787 | -1.4302 |
| **Evi1** | 1.1814 | 1.4661 | 1.1549 | -1.2695 |
| **Fkbp1b** | *-2.0958* | **2.9241** | -1.9069 | *-5.5761* |
| **Fos** | *-2.3158* | **6.4666** | *-3.0743* | *-19.8801* |
| **Fst** | *-2.4360* | **5.9587** | *-2.3591* | *-14.0573* |
| **Gdf1** | *-3.4617* | 1.5922 | 1.4915 | -1.0675 |
| **Gdf2** | 1.9419 | -1.4671 | 1.1382 | 1.6699 |
| **Gdf3** | 1.3732 | **5.3815** | 1.6503 | *-3.2609* |
| **Gdf5** | 1.9419 | -1.4671 | 1.1382 | 1.6699 |
| **Gdf6** | 1.9419 | 2.7378 | **5.2954** | 1.9342 |
| **Gdf7** | *-4.1224* | **8.8581** | **3.7862** | *-2.3396* |
| **Gsc** | 1.9419 | -1.4671 | 1.6595 | **2.4347** |
| **Id1** | *-3.4738* | 1.2303 | *-3.0089* | *-3.7019* |
| **Id2** | **390.3163** | **4.5694** | **142.0987** | **31.0980** |
| **Igf1** | 1.4305 | **2.9201** | 1.5186 | -1.9229 |
| **Igfbp3** | *-7.1975* | **2.8324** | *-2.1042* | *-5.9597* |
| **Il6** | -1.1659 | **2.3603** | *-2.2042* | *-5.2027* |
|  |  |  |  |  |
| **Table S1 (Cont.)** | | | | |
| **Fold Regulation** | | | | |
|  | **Relative to *SPARC+/+*** | | | **Relative to *SPARC-/-*** |
| **Gene Symbol** | ***SPARC+/+* LOS** | ***SPARC-/-*** | ***SPARC-/-* LOS** | ***SPARC-/-* LOS** |
| **Inha** | -1.4444 | 1.6358 | **2.7240** | 1.6653 |
| **Inhba** | *-3.4762* | 1.4419 | *-2.2883* | *-3.2995* |
| **Inhbb** | *-9.0223* | 1.4231 | -1.5318 | *-2.1799* |
| **Itgb5** | *-2.8709* | 1.4845 | -1.9852 | *-2.9470* |
| **Itgb7** | **3.0791** | **2.0069** | 1.1390 | -1.7620 |
| **Jun** | -1.8954 | 1.2675 | 1.4179 | 1.1186 |
| **Junb** | -1.0292 | **8.0055** | **12.9938** | 1.6231 |
| **Lefty1** | 1.9650 | -1.4600 | -1.3007 | 1.1225 |
| **Ltbp1** | -1.0313 | 1.2535 | 1.2006 | -1.0441 |
| **Ltbp2** | -1.1929 | 1.4479 | 1.4781 | 1.0208 |
| **Ltbp4** | -1.0313 | 1.0703 | 1.0380 | -1.0311 |
| **Myc** | *-2.3612* | **2.0111** | *-2.5602* | *-5.1488* |
| **Nbl1** | 1.2759 | **2.4351** | **2.2203** | -1.0968 |
| **Nodal** | 1.9419 | **3.7166** | 1.1382 | *-3.2654* |
| **Nog** | 1.9419 | -1.3557 | 1.1382 | 1.5430 |
| **Nr0b1** | 1.9419 | -1.4671 | 1.1382 | 1.6699 |
| **Pdgfb** | *-4.1916* | **3.3566** | 1.0576 | *-3.1739* |
| **Plat** | *-4.6767* | 1.0504 | -1.2069 | -1.2677 |
| **Plau** | *-4.3575* | **3.6100** | *-2.3090* | *-8.3354* |
| **Runx1** | -1.2566 | 1.2675 | 1.6299 | 1.2859 |
| **Serpine1** | *-2.3384* | 1.1728 | *-5.7328* | *-6.7237* |
| **Smad1** | *-4.2442* | **2.4829** | *-2.5075* | *-6.2258* |
| **Smad2** | *-9.5566* | 1.1111 | -1.2161 | -1.3512 |
| **Smad3** | -1.3972 | 1.7826 | 1.4023 | -1.2713 |
| **Smad4** | *-5.6197* | **6.0629** | -1.1690 | *-7.0874* |
| **Smad5** | 1.0486 | 1.1196 | -1.5684 | -1.7559 |
| **Smurf1** | -1.3127 | 1.4784 | 1.4547 | -1.0162 |
| **Sox4** | *-6.7388* | 1.8417 | 1.4874 | -1.2382 |
| **Stat1** | 1.1348 | -1.4379 | -1.9456 | -1.3531 |
| **Tdgf1** | 1.9419 | -1.4671 | 1.1382 | 1.6699 |
| **Tgfb1** | *-3.2344* | 3.2087 | 1.2779 | *-2.5110* |
| **Tgfb1i1** | -1.5310 | 1.7789 | 2.0630 | 1.1597 |
| **Tsc22d1** | *-2.0929* | 1.2483 | -1.1105 | -1.3863 |
| **Tgfb2** | *-2.1924* | 1.7715 | **2.2065** | 1.2455 |
| **Tgfb3** | *-2.6935* | **2.1053** | 1.3147 | -1.6013 |
| **Tgfbi** | *-2.2792* | **5.5328** | *-2.5320* | *-14.0087* |
| **Tgfbr1** | -1.2393 | 1.1950 | 1.2064 | 1.0096 |
| **Tgfbr2** | *-3.1372* | **3.8185** | 1.2868 | *-2.9675* |
| **Tgfbr3** | *-3.8974* | **6.5933** | 1.1311 | *-5.8290* |
| **Tgfbrap1** | 1.3191 | *-4.4816* | -1.2271 | **3.6522** |
