# Supplementary material for: Losartan Slows Pancreatic Tumor Progression and Extends Survival of SPARC-Null Mice by Abrogating Aberrant TGFβ Activation
Source: PLoS One. 2012 Feb 14;7(2):e31384. doi: 10.1371/journal.pone.0031384 (PMC3279359; doi:10.1371/journal.pone.0031384)
Supplement: Table S2 — List of antibodies. (DOCX) [file pone.0031384.s004.docx]

| **Table S1** | | | | | | | | | | | |
| --- | --- | --- | --- | --- | --- | --- | --- | --- | --- | --- | --- |
| **Antigen** | **Antibody Clone or Catalog # (Ref)** | **Species** | **Source** | **Frozen** | **Methyl Carnoy's** | **Formalin** | **Retrieval Citrate** | **Retrieval PK Digest** | **DAB** | **AEC** | **FL** |
| **CD11b** | **M1/70** | **Rat** | **AbD Serotec, Raleigh, NC** | **√** |  |  |  |  |  |  | **√** |
| **CD163** | **M-96** | **Rabbit** | **Santa Cruz Biotechnology Inc., Santa Cruz, CA** | **√** |  |  |  |  |  |  | **√** |
| **CD31** | **ab28364** | **Rabbit** | **Abcam Inc., Cambridge, MA** | **√** |  |  |  |  |  |  | **√** |
| **Collagen I (a1), C-terminal telopeptide** | **LF-67 (1,2)** | **Rabbit** | **Dr. Larry Fisher, NIH/NIDCR Matrix Biology Unit** |  |  | **√** | **√** |  |  |  | **√** |
| **Collagen I (a2)** | **46425** | **Rabbit** | **Developed in House** |  |  | **√** | **√** |  |  |  | **√** |
| **Collagen IV** | **AB756P** | **Rabbit** | **Chemicon International Inc., Temecula, CA** |  | **√** |  |  |  |  |  | **√** |
| **Decorin** | **AF1060** | **Goat** | **R&D Systems, Minneapolis, MN** |  | **√** |  |  |  | **√** |  |  |
| **Endomucin** | **V.7C7** | **Rat** | **Santa Cruz Biotechnology Inc., Santa Cruz, CA** |  | **√** |  |  |  |  |  | **√** |
| **F4/80** | **Cl:A3-1** | **Rat** | **AbD Serotec, Raleigh, NC** |  | **√** |  |  | **√** |  |  | **√** |
| **Fibronectin** | **DP3060** | **Rabbit** | **Acris Antibodies, Hiddenhausen, Germany** |  | **√** |  |  |  |  | **√** |  |
| **iNOS** | **NB120-15323** | **Rabbit** | **Novus Biologicals, Littleton, CO** | **√** |  |  |  |  |  |  | **√** |
| **Laminin** | **AHP420** | **Rabbit** | **AbD Serotec, Raleigh, NC** |  | **√** |  |  |  |  | **√** |  |
| **Ly-6G/Ly-6C (GR-1)** | **RB6-8C5** | **Rat** | **Biolegend, San Diego, CA** | **√** |  |  |  |  |  |  | **√** |
| **Mac-3** | **M3/84** | **Rat** | **PharMingen/BD Biosciences, San Jose, CA** |  | **√** |  | **√** |  |  |  | **√** |
| **Mannose Receptor (MMR) (CD206)** | **MR5D3** | **Rat** | **Biolegend, San Diego, CA** | **√** |  |  |  |  |  |  | **√** |
| **Myeloperoxidase (MPO)** | **HP9048** | **Rabbit** | **Hycult Biotechnology, Uden, Netherlands** | **√** |  |  |  |  |  |  | **√** |
| **NG2 chondroitin sulfate proteoglycan** | **AB5320** | **Rabbit** | **Chemicon International Inc., Temecula, CA** | **√** |  |  |  |  |  |  | **√** |
| **Pan Endothelial Cell Marker** | **Meca-32 (*3*)** | **Rat** | **Developmental Studies Hybridoma Bank, University of Iowa, Iowa City, IA** |  | **√** |  |  |  |  |  | **√** |
| **Pan Reticular Fibroblast Marker** | **ER-TR7** | **Rat** | **Biogenesis Ltd, Poole, UK** |  | **√** |  | **√** |  |  |  | **√** |
| **Smooth Muscle Actin (SMA)** | **RB-9010** | **Rabbit** | **Lab Vision, Fremont, CA** |  | **√** |  |  |  |  |  | **√** |
| **VEGFR2** | **RAFL-2 (*4*)** | **Rat** | **Dr. P. Thorpe, UT Southwestern Medical Center, Dallas, TX** | **√** |  |  |  |  |  |  | **√** |
| **VEGF:VEGFR Complex** | **Gv39M- Biotinylated (*5*)** | **Mouse** | **Purified in House** | **√** |  |  |  |  |  |  | **√** |
